# Prophylaxis of Syphilis

**Published:** 1852-02

**Authors:** 


*Prophylaxis of Syphilis.*—Dr. Langlebert announced to the Academy that he had discovered a substance capable of destroying the effects of the syphilitic virus. This preservative consists of a strong alcoholic solution of soap, having an excess of alkali. Dr. Langlebert had performed several experiments by inoculating with the poison of chancres, and then applying his antidote within about five minutes afterwards. The effects of the virus had in every case disappeared.—*Lon. Med. Gaz.*

